# Treatment patterns and costs of care for patients with relapsed and refractory Hodgkin lymphoma treated with brentuximab vedotin in the United States: A retrospective cohort study

**DOI:** 10.1371/journal.pone.0180261

**Published:** 2017-10-09

**Authors:** Shelagh M. Szabo, Ishan Hirji, Karissa M. Johnston, Ariadna Juarez-Garcia, Joseph M. Connors

**Affiliations:** 1 Broadstreet HEOR, Vancouver, British Columbia, Canada; 2 Bristol-Myers Squibb, Princeton, New Jersey, United States of America; 3 BC Cancer Agency Centre for Lymphoid Cancer, Vancouver, British Columbia, Canada; Katholieke Universiteit Leuven Rega Institute for Medical Research, BELGIUM

## Abstract

**Objectives:**

Although brentuximab vedotin (BV) has changed the management of patients with relapsed or refractory Hodgkin lymphoma (RRHL), little information is available on routine clinical practice. We identified treatment patterns and costs of care among RRHL patients in the United States (US) treated with BV.

**Methods:**

A retrospective observational study of adults initiating BV for RRHL from 2011–2015, with ≥6 months of data prior to and following BV initiation, was conducted. Treatments were classified based on dispensations and chemotherapy administration. Median total and monthly costs were estimated based on all-cause healthcare resource use in 2015 US dollars (USD).

**Results:**

The cohort comprised 289 patients (59% male; mean age at diagnosis, 42 years) with a mean follow-up of 250 weeks. Eleven percent had BV salvage therapy prior to ASCT, and 32% had BV for a relapse post-ASCT. 43% received treatment post-BV, most commonly allogeneic stem cell transplant (SCT) and bendamustine (both 10.2%). Median (IQR) total costs from BV initiation to censoring were 294,790 (142,110–483,360) USD; and were highest among those treated with BV prior to ASCT (up to 421,900 [300,940–778,970] USD). Median monthly costs were almost 20,000 USD per month, and up to 25,000 USD per month among those with BV and ASCT. Medications were the greatest driver of median monthly costs.

**Conclusions:**

Median total all-cause costs were almost 300,000 USD, and median monthly costs approximately 20,000 USD, per patient treated. Patients requiring treatment following BV continue to incur high costs, highlighting the economic burden associated with managing patients in the RRHL setting.

## Introduction

Hodgkin lymphoma (HL) is a neoplasm of the lymphatic system. In the early stages, individuals afflicted are typically asymptomatic and present with enlarged or hardened lymph nodes, commonly in the cervical and supraclavicular regions.[1, 2] When diagnosed early, while the cancer is still localized, the prognosis of HL tends to be very favorable.[1, 3] Most patients with HL can be cured with first-line multi-agent chemotherapy, with or without radiation; however, a substantial proportion—approximately 10–20%—develop progressive disease despite primary treatment, and require second-line therapy.[4] Of these patients, approximately 50% will be cured, most often with high-dose chemotherapy plus autologous stem cell transplant (ASCT).[4–6] While treatment options for the relatively small population of patients with HL who experience a relapse or are refractory after ASCT were historically quite limited, recent advances are providing new hope for improved outcomes.

Brentuximab vedotin (BV), an antibody–drug conjugate that targets the CD30 protein in classical HL, has changed the management of patients with relapsed or refractory HL (RRHL). Results from clinical trials demonstrate that almost three-quarters of those treated respond to BV, with a median objective response of 11.2 months.[7] BV is indicated for patients with HL who experience a relapse after ASCT, or after the failure of two or more prior multi-agent chemotherapy regimes in patients who are not candidates for ASCT.[8] Additionally, there is evidence emerging for the use of BV among HL patients who are at a high risk of relapse or progression as post-ASCT consolidation,[8, 9] or as a salvage or bridge regimen prior to ASCT.[10, 11] However, limited real-world data exist on the management of patients with RRHL, or their costs of care, in routine clinical practice in the post-BV era.[12] Contemporary estimates are required to help understand the current economic burden of RRHL, and also to provide context for clinical and economic costs and benefits of new treatments for RRHL.

The primary objective of this study was to identify treatment patterns among a cohort of patients with RRHL in the United States (US) treated with BV, prior to and following the initiation of treatment with BV. Secondary objectives included characterizing the population with RRHL who receive treatment with BV; and estimating mean and total direct medical costs among patients with RRHL from time of initiation, or discontinuation, of treatment with BV.

## Methods

### Study design

This retrospective cohort study used data from Truven MarketScan, a large integrated US claims database, to identify treatment patterns and estimate costs of patients with RRHL treated with BV (‘BV RRHL cohort’). The study period was from 1 January 2006 to 30 June 2015.

#### Data source

Data from the 2006 to 2015 Truven MarketScan Commercial and Medicare databases were used, as well as from the 2010 to 2014 Truven MarketScan Medicaid databases (http://truvenhealth.com/markets/life-sciences/products/data-tools/marketscan-databases); all patients in these databases who met the study eligibility requirements were included. The dataset included inpatient admissions and procedures, outpatient visits and procedures, and outpatient prescription records, with associated diagnostic codes, from a large, convenience sample of individuals from the US covered under commercial, Medicare, or Medicaid health insurance plans. The MarketScan Commercial database contains records on approximately 31.3 million individuals covered under a variety of employer-sponsored insurance plans and their dependents; claims data are derived from over 100 insurers.[13] The MarketScan Medicare database includes data on healthcare received by approximately 3.1 million retirees with Medicare supplemental insurance paid for by employers, and their Medicare-eligible dependents.[14] The MarketScan Medicaid datasets includes data on healthcare received by 6 million individuals who qualify for Medicaid due to low income or disability, from 10 states.[14, 15] These databases provide detailed data for analyses of costs, resource use, and outcomes research.[16–18] However, given that these are administrative claims databases, clinical data from which to directly estimate factors like treatment response were not available. All data were de-identified and compliant with the Health Insurance Portability and Accountability Act (HIPAA) of 1996. Because the Truven MarketScan data are de-identified and are fully HIPAA compliant, and because this study did not involve the collection, use, or transmittal of individually identifiable data, Institutional Review Board review or approval was not required.

### Study sample

The target population for the analysis was patients with RRHL who receive treatment with BV.[7, 19] Patients with RRHL were identified by a dispensation of BV[20] and diagnostic code for HL (by International Classification of Diseases–9th Revision [ICD-9] code 201.X),[20, 21] without a diagnostic code for anaplastic large cell lymphoma (200.6). As a more specific definition of RRHL and to identify a more homogeneous sample, we also focused specifically on the subgroup treated with both BV and ASCT.[19]

For the primary analysis, a minimum of 6 months of enrollment data prior to and after the index date was required (BV enrollment cohort), to ensure the availability of sufficient follow-up data for each patient to observe outcomes of interest. The requirement for ≥6 months of enrollment data was relaxed in a sensitivity analysis that included all patients, to maximize the sample size of patients with RRHL.

The relationship between the study cohorts is illustrated in S1 Fig; including an illustration of the relationship between the RRHL subpopulation and the larger classical HL population. A control group without HL was not included.

### Classifying lines of therapy

Treatment patterns were classified according to individual or multi-agent therapies observed within successive lines of therapy. Individual treatment regimens observed were classified according to line of therapy, using the algorithm below that was developed through iterative clinical consultation. Lines of therapy were considered according to the timing of their use relative to the timing of the administration of BV and/or ASCT.

Individual and combination chemotherapies were identified by Healthcare Common Procedural Coding System (HCPCS) codes and National Drug Codes (NDCs),[20, 22, 23] specific to the agents considered (S1 Table). To identify a line of chemotherapy, the service date for the first claim for any chemotherapy occurring within an individual’s enrollment data was captured. Any other therapies occurring during or within 60 days of the dates of the first regimen identified were considered to occur in combination with first-line therapy. Patients were assumed to continue on a line of therapy until a) the initiation of a new therapy greater than 30 days after the cessation of a first-line therapy; or b) the initiation of a known second-line therapy among those treated with a known first-line therapy (Adriamycin [doxorubicin], bleomycin, vinblastine, and dacarbazine [ABVD]; bleomycin, etoposide, Adriamycin, cyclophosphamide, Oncovin [vincristine], procarbazine, prednisone [BEACOPP]; Stanford V regimen; or cyclophosphamide, vincristine, procarbazine, prednisone [C-MOPP]; S1 Table). In addition to a line of therapy ending with the appearance of a new medication, inpatient death, de-enrollment in the health insurance plan, or reaching the end of the study period (30 June 2015) was also used to classify the end of a line of therapy. Patients who died during an inpatient admission, ceased enrollment, or reached the end of the study period were considered censored at the time of those events.

Please see S1 Table—HL-specific medications identified in the Truven MarketScan databases.

This process was repeated for each subsequent line of therapy. As treatment of HL can be highly individualized, multi-agent therapies were classified if all components were administered within a 60-day period, or if only a partial set (one or more) of the components of the multi-agent therapy were administered.

The occurrence of selected procedures (radiation, allogeneic stem cell transplant [allo-SCT], and ASCT) were also identified by HCPCS codes,[20, 22] and tabulated within each line of therapy. For allo-SCT and ASCT, these were classified within an individual line of therapy if they occurred within up to 8 months of the cessation of the latest date of a chemotherapy within that line of therapy (to account for time required for radiation or other therapy, as necessary, prior to SCT); and for radiation, if they occurred within 75 days of that line of therapy.

Because of incomplete follow-up data, despite the requirement for ≥6 months of enrollment data prior to and after BV initiation, some treatments for HL may have occurred prior to the start of the observation period for a given patient.

### Outcomes and analysis

The analyses conducted are largely descriptive. Categorical variables are presented as counts with percentages of patients in each category. Continuous variables are summarized by means, standard deviations (SD), 95% confidence intervals (CI), medians, and interquartile ranges (IQR). All outcomes were summarized for the entire BV enrollment cohort, and separately according to clinical subgroup; for those with BV in a line of therapy prior to the line in which patients received ASCT [BV in the line prior to ASCT], BV post-ASCT relapse (e.g. BV in a line of therapy after ASCT), BV maintenance after ASCT (e.g. BV within the same line as, but occurring after ASCT), BV salvage therapy prior to ASCT (e.g. BV within the same line as, but occurring prior to, ASCT), and BV without observed ASCT.

The age, sex, year of first recorded diagnosis, and primary payer were tabulated. Demographic characteristics were summarized. The mean (SD) and median (IQR) duration of follow-up from time of BV initiation to censoring was estimated, and considered separately for the time periods prior to BV initiation, while receiving BV, and after BV discontinuation. Follow-up time was defined from the time of BV initiation until censoring (at death or de-enrollment from the plan).

To summarize treatment patterns, the frequency of patients receiving BV (with ASCT, if applicable), according to line of therapy, was estimated. Duration on and cycles of BV were calculated. The number (and percentage) of patients with observed lines of therapy prior to, concurrent with, or after the line of therapy including BV was tabulated, and the most frequently utilized regimens (in terms of percentages of users, for therapies administered to ≥1.5%) calculated.

Median (IQR) total and monthly all-cause costs were evaluated based on underlying healthcare resource utilization. Total costs were calculated from the first observed RRHL treatment, to the end of an individual’s available follow-up; for monthly costs, a person-time denominator was employed to account for variability in the duration of follow-up. Healthcare costs were measured by data within financial fields on administrative claims within the databases, and included the gross covered payments for all healthcare services or products (i.e., the amount eligible for payment after applying pricing guidelines such as fee schedules and discounts, but including deductibles, copayments, and coordination of benefits). Costs were classified as inpatient-, outpatient-, or medication-related costs based on designations contained within the MarketScan databases with one exception; chemotherapy-related costs were reclassified from outpatient costs (if applicable) to medication costs. Median total costs do not necessarily reflect lifetime costs, as some individuals were censored by loss to follow-up. In the available data, reason for leaving the plan is not known in detail; thus, it is not possible to differentiate death from loss to follow up, and a detailed assessment of censoring is not feasible. Note, for monthly and total costs, while both means and medians were calculated, median estimates only are presented here, for simplicity of data presentation and to account for skew in the underlying costs data.

All costs were updated to 2015 US dollars (USD). All analyses were performed in R for Windows; results were exported to Microsoft Excel.

### Sensitivity analysis

Two sensitivity analyses were conducted to assess the impact of key study design assumptions and population features on outcomes for the BV RRHL cohort. First, a sensitivity analysis was performed where the requirement for ≥6 months of enrollment data prior to and after initiation of BV was relaxed. This analysis was performed in order to understand how generalizable the results from the more highly selected sample with minimum enrolment criteria were to the broader patient population included in the MarketScan datasets. Second, patients who only ever had one dose of BV were excluded, to avoid biasing estimates of costs by including individuals who may have had less resource use than the average patient with BV (due to their treatment being stopped early). Two sets of parameters were compared between the base case and each of the sensitivity analysis scenarios: the proportion treated prior to and after BV, and mean (95% CI) monthly costs.

## Results

Of 666 patients who were ever treated with BV, 122 were excluded for anaplastic large cell lymphoma. Of the remaining 544, 289 had ≥6 months of enrollment data before and after BV initiation, and were therefore eligible for inclusion in the cohort. The mean (SD) age at diagnosis was 42.3 (17.2) years, 58.5% were male, and 66.8% had coverage under commercial insurance plans (Table 1). Of the 289 patients, 33 (11.4%) had BV salvage prior to ASCT, 91 (31.5%) had BV post-ASCT relapse, and 156 (54.0%) had BV without an observed ASCT; as only 1 (0.3%) had BV maintenance therapy after ASCT, and 8 (2.8%) had BV in the line prior to ASCT, results for these subgroups should be interpreted with caution. Patients who had ever received BV and ASCT tended to be younger (mean age, 35.9 years vs. 47.8 years for those who were never observed to receive ASCT) and were more likely to have commercial insurance (73.7% vs. 60.9% for those who were never observed to have received ASCT).

**Table 1:** Demographic characteristics of the 289 patients in the MarketScan database with ≥6 months of enrollment before and after BV initiation.

	All BV (n = 289)	BV in the line prior to ASCT (n = 8)	BV salvage pre-ASCT (n = 33)	BV post-ASCT relapse (n = 91)	BV with no observed ASCT (n = 156)
**Age at diagnosis, years**					
Mean (SD)	42.3 (17.2)	37.4 (10.1)	35.8 (13.0)	35.6 (12.8)	47.8 (18.6)
Median (IQR)	40.0 (28.0–54.0)	40.0	34.0	32.0	46.0
		(32.8–41.5)	(26.0–43.0)	(25.5–45.5)	(32.0–64.5)
**Male, n (%)**	169 (58.5)	8 (100.0)	18 (54.5)	54 (59.3)	88 (56.4)
**Year of diagnosis, n (%)**					
2006–2011	133 (46.0)	3 (37.5)	11 (33.3)	59 (64.8)	60 (38.5)
2012–2016	135 (46.7)	5 (62.5)	22 (66.7)	30 (33.0)	77 (49.4)
**Primary payer, n (%)**					
Medicare	39 (13.5)	0 (0.0)	1 (3.0)	2 (2.2)	36 (23.1)
Medicaid	57 (19.7)	1 (12.5)	6 (18.2)	25 (27.5)	25 (16.0)
Commercial	193 (66.8)	7 (87.5)	26 (78.8)	64 (70.3)	95 (60.9)
**Prior ASCT, n (%)**	92 (31.8)	0 (0.0)	0 (0.0)	91 (100.0)	0 (0.0)

ASCT, autologous stem cell transplant; BV, brentuximab vedotin; IQR, interquartile range; SD, standard deviation.

The mean (SD) duration of enrollment for the BV cohort was 251.8 (126.6) weeks. The mean (SD) duration prior to initiation of BV was 180.6 (118.9) weeks, post-BV discontinuation was 48.5 (36.5) weeks, and while on BV was 22.7 (23.8) weeks (S2 Table); corresponding to a mean of 7.6 cycles of BV. Mean duration of enrollment was similar between the BV subgroups (ranging from 249.7 weeks for BV post-ASCT relapse, to 277.9 weeks for BV in the line prior to ASCT). However, the mean time on BV differed by subgroup; mean cycles of BV were 2.6 (BV in the line prior to ASCT), 5.7 (BV salvage therapy prior to ASCT), 7.3 (BV post-ASCT relapse), and 8.4 (BV without observed ASCT). The one patient who underwent BV maintenance after ASCT had 121.9 weeks of follow-up available (including 23.6 weeks receiving BV therapy).

Please see S2 Table—Time from plan enrollment to BV initiation, and from BV initiation to censoring, in weeks.

**Fig 1:**
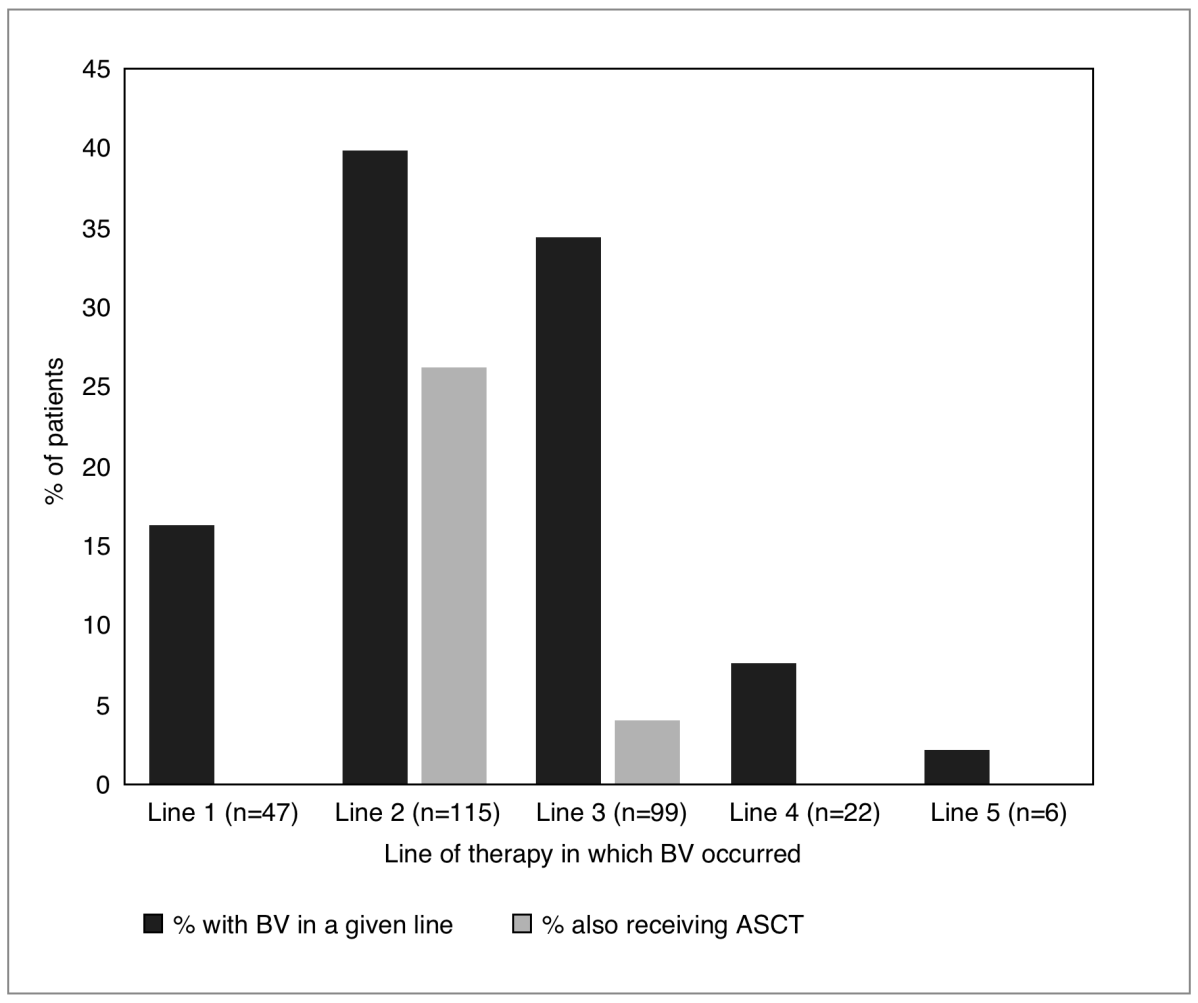
Timing of treatment with BV, and co-occurrence with ASCT, within observed lines of therapy.

**Fig 2:**
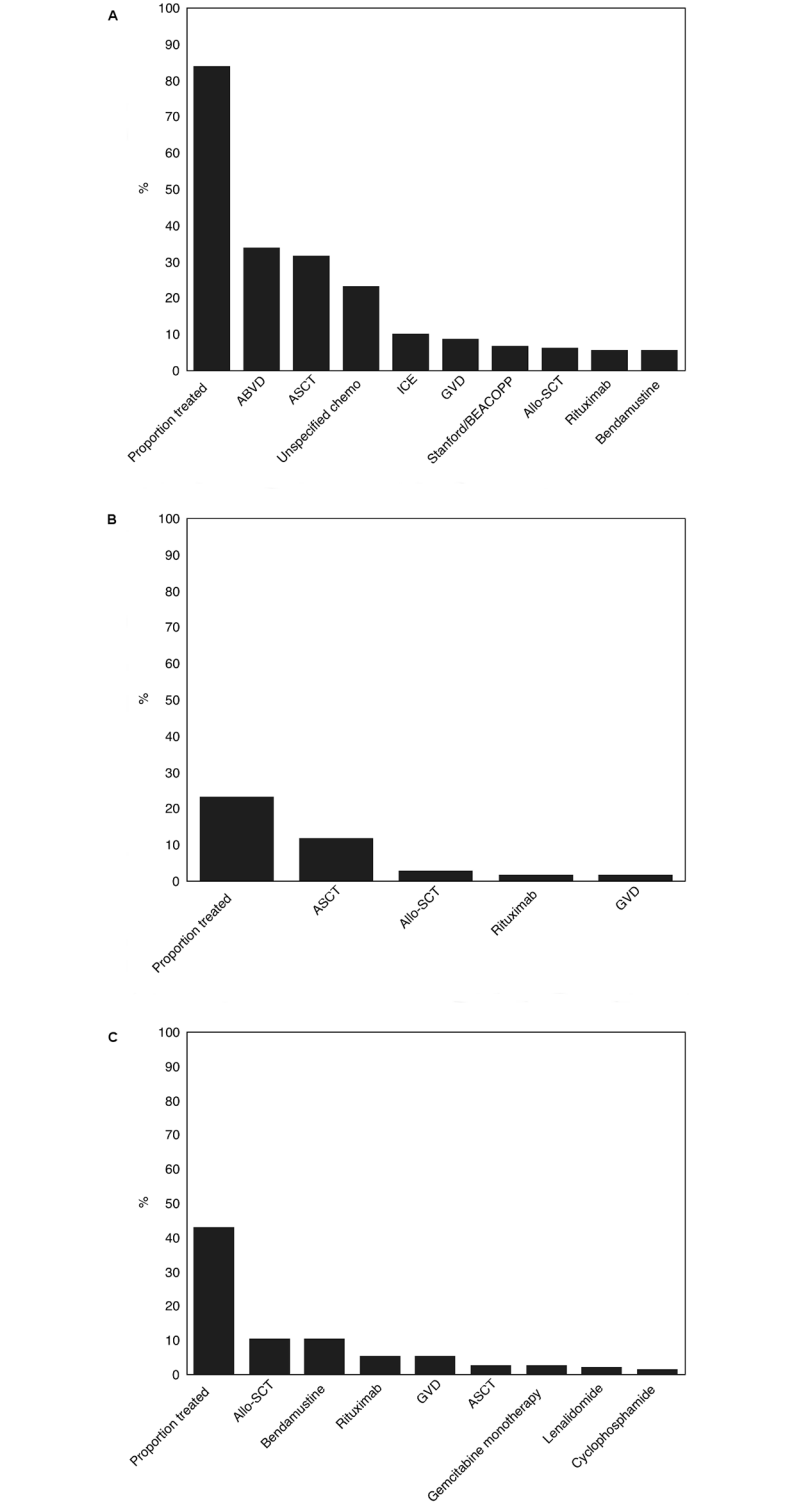
Frequency of therapies occurring a) in a line of therapy prior to BV; b) within the same line of therapy as BV; and c) in a line of therapy after the line of therapy including BV.

Most patients received treatment with BV within their second (40.0%) or third (34.3%) observed line of therapy (Fig 1). The frequency of observed therapies in lines of therapy prior to, concurrent with, and following the line of therapy in which BV was initiated is presented in Fig 2. A prior therapy was observed in 83.7% of patients, the most frequent of which was ABVD (33.9%). A total of 23.2% of patients received another therapy concurrent with BV, the most frequent of which was ASCT (11.8%). Finally, 42.9% received therapies after the line in which they had received BV, the most frequent of which were allo-SCT and bendamustine (10.4% each). Forty-eight individuals (52.7%) who received BV after a relapse post-ASCT went on to receive another subsequent therapy (most commonly allo-SCT and bendamustine, 16.5% each); as did 9 (27.3%) individuals who had BV salvage therapy prior to ASCT (most commonly allo-SCT, 6.1%), 59 (37.8%) individuals with BV without an observed ASCT (most commonly bendamustine, 8.3%), and 1 (12.5%) individual who had BV in the line prior to ASCT (allo-SCT, 12.5%).

Median (IQR) monthly costs, according to time (during and after BV; including while receiving BV vs. after BV discontinuation), are presented in Table 2, as well as according to BV subgroup. Median (IQR) monthly costs for the overall group during and after BV treatment were 19,460 (11,350–33,000) USD, and were 24,620 (14,720–37,540) USD while receiving BV and 15,800 (5,700–32,330) USD after BV discontinuation. Of all the subgroups considered, median (IQR) monthly costs among patients treated with BV prior to ASCT were highest (25,050 [16,530–38,2200] USD for those treated with BV in the line prior to ASCT, and 24,670 [11,880–40,060] USD for those with BV salvage therapy prior to ASCT), and less among those with BV post-relapse (20,040 [11,510–34,220]) or among those without an observed ASCT (18,320 [10,690–32,260]). Median monthly costs were generally higher in the period while receiving BV, compared to after BV discontinuation. Monthly costs among the one patient with BV maintenance therapy after ASCT were high (40,640 USD).

**Table 2:** Median (IQR) monthly all-cause costs (USD), according to timing with respect to BV treatment.

	During and post-BV		While receiving BV		After BV discontinuation	
	Median	IQR	Median	IQR	Median	IQR
**All BV (n = 289)**	19,460	11,350–33,000	24,620	14,720–37,540	15,800	5,700–32,330
**BV in the line prior to ASCT (n = 8)**	25,050	16,530–38,220	12,990	5,910–33,970	21,980	16,740–38,110
**BV salvage therapy prior to ASCT (n = 33)**	24,670	11,880–40,060	28,980	18,350–49,930	17,800	9,480–41,040
**BV post-ASCT relapse (n = 91)**	20,040	11,510–34,220	25,960	16,360–37,370	16,750	5,460–32,780
**BV without observed ASCT (n = 156)**	18,320	10,690–32,260	23,130	12,920–35,200	14,160	4,810–30,610

ASCT, autologous stem cell transplant; BV, brentuximab vedotin; CI, confidence interval; HL, Hodgkin lymphoma; IQR, interquartile range.

Medications were the greatest driver of median monthly costs for the entire cohort, for those with BV post-ASCT relapse, for those without observed ASCT, and for the single individual with BV maintenance therapy after ASCT; median medication costs were more than three-fold higher than median inpatient or outpatient costs in all these subgroups (Table 3). Inpatient costs were the greatest driver for those with BV in a line prior to ASCT, with median costs approximately 50% higher than median medications or outpatient costs; and inpatient and medication costs contributed almost equally for those with BV salvage therapy prior to ASCT.

**Table 3:** BV enrollment cohort (N = 289): median (IQR) monthly all-cause costs x cost type, according to timing with respect to BV initiation.

	During and post-BV		While receiving BV		After BV discontinuation	
	Median	IQR	Median	IQR	Median	IQR
**All BV (n = 289)**						
** Medication**	10,230	4,800–16,710	21,710	10,260–30,830	4,550	1,990–11,080
** Outpatient**	2,780	1,270–5,690	1,530	590–3,700	2,640	1,050–6,100
** Inpatient**	1,530	0–8,100	0	0–0	660	0–9,100
**BV in the line prior to ASCT (n = 8)**						
** Medication**	6,440	3,390–13,280	9,820	2,070–29,080	4,590	3,100–6,670
** Outpatient**	6,300	3,140–9,290	2,710	220–5,070	4,940	2,750–9,070
** Inpatient**	9,840	8,130–17,750	0	0–0	11,970	8,960–18,940
**BV salvage therapy prior to ASCT (n = 33)**						
** Medication**	7,090	3,340–16,710	24,660	14,940–35,770	3,910	1,320–7,130
** Outpatient**	3,210	1,810–8,050	2,550	640–6,380	3,000	1,460–6,860
** Inpatient**	7,250	4,300–15,930	0	0–0	7,600	1,280–21,550
**BV post-ASCT relapse (n = 91)**						
** Medication**	10,090	5,370–16,850	21,880	11,860–30,920	4,410	1,900–11,040
** Outpatient**	3,080	1,420–6,110	1,580	550–4,000	2,700	1,260–6,280
** Inpatient**	1,820	0–11,400	0	0–0	570	0–10,010
**BV without observed ASCT (n = 156)**						
** Medication**	11,280	5,020–16,670	21,030	9,660–30,010	4,850	2,130–11,380
** Outpatient**	2,330	1,040–4,990	1,360	610–3,230	2,100	880–5,270
** Inpatient**	740	0–3,950	0	0–0	0	0–5,050

Allo-SCT, allogeneic stem cell transplant; ASCT, autologous stem cell transplant; BV, brentuximab vedotin; CI, confidence interval; HL, Hodgkin lymphoma; IQR, interquartile range.

Median (IQR) total costs (from first observed RRHL therapy to the end of an individual’s follow-up), according to time (during and after BV; including while receiving BV vs. after BV discontinuation), are presented in Table 4, as well as according to BV subgroup. Median (IQR) total costs for the overall group during and after BV treatment were 294,790 (142,110–483,360) USD, and were 86,500 (27,510–212,090) USD while receiving BV and 98,580 (33,580–279,990) USD after BV discontinuation. Consistent with the trends observed for mean monthly costs, of all the subgroups considered, median (IQR) total costs among patients treated with BV prior to ASCT were highest (421,900 [300,940–778,970] USD for those treated with BV in the line prior to ASCT, and 352,210 [212,180–509,870] USD for those with BV salvage therapy prior to ASCT), and less among those with BV post-relapse (331,380 [145,660–486,380]) or among those without an observed ASCT (247,890 [115,860–461,490]).

**Table 4:** Median (IQR) total (between all-cause costs (USD), according to timing with respect to BV treatment.

	During and post-BV		While receiving BV		After BV discontinuation	
	Median	IQR	Median	IQR	Median	IQR
**All BV (n = 289)**	294,790	142,110–483,360	86,500	27,510–212,090	98,580	33,580–279,990
**BV in the line prior to ASCT (n = 8)**	421,900	300,940–774,970	28,610	4,270–118,400	410,950	190,270–648,620
**BV salvage therapy prior to ASCT (n = 33)**	352,210	212,180–509,870	56,350	31,470–186,170	205,130	116,860–312,810
**BV post-ASCT relapse (n = 91)**	331,380	145,660–486,380	92,160	41,880–223,350	100,350	40,850–337,930
**BV without observed ASCT (n = 156)**	247,890	115,860–461,490	91,750	26,170–208,710	67,890	27,410–224,430

ASCT, autologous stem cell transplant; BV, brentuximab vedotin; CI, confidence interval; HL, Hodgkin lymphoma; IQR, interquartile range.

In the sensitivity analysis, removing the requirement for data prior to and following BV enrollment resulted in a sample size of 534 patients, with a lower frequency of treatment observed either prior to (70% vs. 83%) or following (30% vs. 43%) BV initiation, compared with the base case. Removing the requirement for data prior to and following BV treatment also increased the sample size in the ‘BV consolidation post-ASCT’ subgroup from 1 to 4 patients. Excluding the 31 patients who only ever had a single dose of BV had very little impact on the percentages of patients with treatment observed prior to and following BV (84% vs. 83% in the base case for treatment prior to BV; 43% vs. 43% in the base case for treatment after BV).

Median (IQR) monthly costs, among all patients and according to subgroup, for the base case and each of the sensitivity analysis scenarios, are presented in Table 5. Removing the requirement for data prior to and following BV enrollment had a slight impact on median (IQR) monthly costs, which were 19,460 (11,350–33,000) USD in the base case and 23,090 (11,880–40,640) USD in the sensitivity analysis. Higher costs are likely related to fewer months of follow-up per patient over which to disaggregate the high treatment costs of BV. The sensitivity analysis to remove patients who only ever had a single dose of BV had minimal impact on median monthly costs.

**Table 5:** Median (IQR) monthly costs (USD), under the base case, and for each of the sensitivity and subgroup analysis scenarios.

	Base case BV enrolment cohort (n = 289)			Full BV cohort (n = 534)			Single dose excluded (n = 258)		
	n	Median	IQR	n	Median	IQR	n	Median	IQR
**All BV (n = 289)**	289	19,460	11,350–33,000	534	23,090	11,880–40,640	258	19,950	11,760–32,990
**BV in the line prior to ASCT (n = 8)**	8	25,050	16,530–38,220	11	18,890	13,090–27,430	6	20,750	14,820–25,500
**BV salvage therapy prior to ASCT (n = 33)**	33	24,670	11,880–40,060	46	25,250	13,920–44,720	30	24,890	12,180–39,900
**BV consolidation post-ASCT (n = 1)**	1	40,640	40,640–40,640	4	42,300	36,150–44,700	1	40,640	40,640–40,640
**BV post-ASCT relapse (n = 91)**	91	20,040	11,510–34,220	119	22,310	13,260–36,250	79	22,260	11,890–35,460
**BV without observed ASCT (n = 156)**	156	18,320	10,690–32,260	354	18,320	10,690–32,260	142	18,580	11,590–31,870

Allo-SCT, allogeneic stem cell transplant; ASCT, autologous stem cell transplant; BV, brentuximab vedotin; CI, confidence interval; HL, Hodgkin lymphoma.

## Discussion

In this study using US administrative claims data, treatment patterns and health resource utilization among the subpopulation of patients with classical HL treated with BV for RRHL, were characterized. Members of the core cohort were required to have ≥6 months of enrollment before and after BV initiation, to increase the likelihood of observing key treatments and outcomes of interest. On average, patients had over 250 weeks of data available for analysis; including 180 weeks prior to BV initiation and almost 50 weeks after BV discontinuation. Eighty-four percent had a record of a prior HL therapy before BV, and BV was most frequently observed to be administered within the second and third lines of therapy. Eleven percent were observed to have BV therapy prior to ASCT, and 32% had BV therapy after a relapse of ASCT; only one patient was observed with BV maintenance therapy in the same line but after ASCT. Finally, 43% went on to a subsequent line of therapy after treatment with BV and/or ASCT; which were most frequently therapy with bendamustine or allo-SCT, across treatment groups.

Median total all-cause costs, from time of first observed treatment for RRHL to censoring, were almost 300,000 USD per patient treated. Median total costs for patients treated with both ASCT and BV ranged from over 330,000 USD (for those with BV post-ASCT relapse) to over 420,000 (for those with BV in the line prior to ASCT). As expected, median costs among those who were treated with BV but without an observed ASCT were the lowest, at approximately 250,000 USD per patient. The trend was similar for monthly costs; median all-cause medical costs were almost 20,000 USD per month, ranged from 20,000 to 25,000 USD per month among those with BV and ASCT, and were 18,000 USD per month among those with BV without observed ASCT. Medication costs were the largest component of medical costs, overall and for most subgroups (except for those with BV in the line prior to ASCT). These findings are important, as contemporary evidence on the treatment and costs of caring for patients with RRHL in routine clinical practice in the post-BV era.[12]

The findings from the current study provide additional evidence on the treatment of RRHL with BV in clinical practice. Two recent systematic reviews by Zinzani et al., have presented syntheses of clinical outcomes of patients with RRHL treated with BV under a Named Patient Program in approximately 60 countries (outside of the US and Canada).[24, 25] Those observational studies reported comparable efficacy and safety associated with BV use in real-world settings, as have been observed in the pivotal BV trials.[26] While outcomes of interest differed between those publications and the present study, available baseline demographic data (mean age and proportion male) were consistent between the cohorts. Zinzani et al., reported at least some proportion of patients (up to 30–40%, depending upon the study) were not treated with ASCT prior to BV. In the present study, we observed a slightly higher percentage of patients treated with ASCT after BV (13.5%), and slightly lower percentage of patients treated with allo-SCT after BV (10.4%) compared to that observed in the review by Zinzani et al. (10–12% for ASCT, and 19% for allo-SCT). The treatment pattern findings of the present study are also broadly consistent with data from a smaller retrospective study by Shao et al.,[27] wherein 50% of patients received treatment after BV.

The findings from the current study also provide context for existing evidence of the economic burden of RRHL. Chemotherapy and transplant costs are key contributors to the economic burden, particularly for patients with RRHL. In a previous retrospective study, Yasenchak et al.[12] estimated the mean total cost at 21,956 (USD 2007) among adult HL patients treated in first-line, 77,219 USD among those treated in second-line, and 59,442 USD among those treated in third-line. That study was from the pre-BV era, however, and until the present study, more contemporary estimates that account for costlier therapies (such as BV, rituximab, and allo-SCT) had not yet been presented. Another study, by Hansen et al.,[28] also from the pre-BV era, estimated the mean (SD) total unadjusted costs for relapsed patients at 401,529 (262,385) (USD 2012) versus 89,709 (105,799) USD for non-relapsed patients. Median total costs per relapsed patient estimated within the present study were approximately 300,000 USD overall; and up to 420,000 USD among the patients comprising the most costly subgroup. Mean total costs, which were tabulated but not presented in this manuscript, were uniformly higher than median costs (as expected); and higher than those estimated by Hansen et al. Those differences would be explained by the difference in time periods and treatments available between the two studies. Shao et al., also estimated mean monthly and total costs among a cohort of patients with RRHL in an abstract presented at the American Society of Hematology 2016. The cohort included in the present study was larger, and costs were higher, than in the study by Shao et al.; however, it is unclear how chemotherapy costs were considered in the study by Shao et al.[29] As the majority of published literature describes costs from the pre-BV era, our results describe important differences in treatment patterns and costs reflecting current treatment offerings. In addition, presentation of results stratified by patient subset based on receipt of ASCT and/or BV, by line of therapy, and by category of resource utilization, allows for a better understanding of cost drivers, and of the relationship between treatments received and corresponding medical costs.

While we had initially considered enrolling an incident cohort of patients with HL and following them to observe the development of RRHL, preliminary feasibility analyses demonstrated that the absolute sample size would be small using this approach.[30] This is not surprising, given issues with continuity of coverage in the US claims datasets.[27] Instead, the study cohort was enrolled at the time of initiation of therapy with BV. While this strategy ensured we had a large sample of patients with RRHL to understand contemporary treatment patterns and costs, it meant reduced visibility for treatments and events occurring prior to a patient’s enrollment in the plan under which they received BV, and also explains why we only observed a prior therapy among 84% of the cohort. This limitation could have also impacted another treatment group of interest: patients treated with BV and ASCT. Although we analyzed outcomes among this group, it is conceivable that we may have missed cases wherein ASCT occurred prior to or after the period of observation for a given patient. On the other hand, as treatment patterns for HL are changing, some patients may now be treated with BV relatively early in their disease course, or prior to ASCT,[31] both of which were situations observed in the present study.

Strengths of the study include that the Truven databases capture integrated data from a very large sample of individuals covered under commercial and Medicare plans, nationwide, and from Medicaid plans from eleven US states. A comprehensive set of codes, consistent with those used by other studies using administrative data in the published literature, were used to identify the exposures and outcomes of interest. Data over a 10-year period were analyzed, allowing for the opportunity to observe long-term follow-up, and primary and subsequent treatments for HL. While not all individuals have full prescription dispensation data available in the Truven databases, the vast majority of treatments of interest for HL are either administered in an outpatient clinic setting, or identifiable by procedural codes if administered to an inpatient, and as such are consistently captured within the available data. Throughout the study, iterative consultations were undertaken with a clinical expert specializing in the treatment of HL, to ensure the face validity and to guide the interpretation of results. No requirement for continuous enrollment prior to study entry was imposed, to avoid any selection bias. As the most recent data on costs in the US are from 2007 to 2011, which was prior to the introduction of BV, these data filled an existing knowledge gap on the economic burden of HL.

As with any retrospective study, the findings were limited by the availability of data and the duration of follow-up of patients within the databases. Administrative claims data are collected for billing, rather than research, purposes. This may cause misclassification as coding may be driven by reimbursement (rather than clinical) factors. Given the sampling frame for the study (a convenience sample of individuals with commercial, Medicare, or Medicaid plans), the study findings may not be reflective of treatment or outcomes for patients who have other types of insurance or those without insurance. The sample included in the Commercial database is thought to be nationally-representative of working-aged and employed individuals in the US.[32, 33] Findings from the Medicare sample would be specific to older adults who had previously been working and now are covered under employer-sponsored Medicare programs; the Medicaid data have the most limited generalizability as they wre available on only approximately half of the overall study period, and from only eleven states. However, excluding the Medicaid patients from the analysis did not materially affect the study findings (*data not shown)*.

As the Truven data were based on claims with limited associated clinical information, algorithms were used to infer line of therapy, and these introduce the potential for misclassification. Given that individuals with intermittent healthcare coverage may be included, healthcare resource use and costs may be underestimated. Costs data available in the dataset reflect insurance payments, and therefore are underestimates for total costs of care, as some costs (denied payments, direct non-medical costs, and costs for uninsured services) would not be included. Specific to medication dispensations, only outpatient dispensations covered by commercial, Medicare, or Medicaid insurance appeared in the database, and no data were available on patterns of actual prescription usage; data on inpatient and outpatient chemotherapies are available, however, through procedural records. While we selected the core cohort and restricted it to patients with at least 6 months of enrollment before and after BV initiation, to increase the likelihood of observing key treatments and outcomes of interest, this eliminated almost half of those treated with BV from the core analyses. These additional cases were included in a sensitivity analysis and the analysis of data from the full cohort yielded higher mean monthly costs; our core approach could therefore be considered conservative. Excluding patients who only ever had one dose of BV had a minimal impact on estimates of the frequency of treatment or costs. Total costs, from time of first observed RRHL treatment to the end of an individual’s follow up, were calculated to help understand the magnitude of the economic burden of RRHL. With the available data, it was not possible to estimate lifetime costs, because these data are not population-based and few individuals would have the required level of follow-up detail; as well as that mortality data were only available for the subset of individuals with an inpatient record of death. Since reason for leaving the plan is not known in detail, it is not possible to differentiate death from loss to follow up, and a detailed assessment of censoring is not feasible. Finally, treatment of patients enrolled in randomized trials would not have been captured.

This retrospective study highlights the high clinical, and associated economic, burden of managing patients in the RRHL setting with currently available therapies. Median total all-cause costs were almost 300,000 USD per patient treated, and median monthly all-cause costs were approximately 20,000 USD. These findings augment the scarce real-world data on outcomes among patients with RRHL, and can be used as benchmarks against which to compare treatment patterns, costs, and outcomes after the introduction of emerging therapies.

## Supporting information

S1 Fig Selection of the study cohorts, Truven MarketScan data 2006–2015. (TIF)

S1 Table HL-specific medications identified in the Truven MarketScan databases. (DOCX)

S2 Table Time from plan enrollment to BV initiation, and from BV initiation to censoring, in weeks. (DOCX)
