# Supplementary material for: Treatment patterns and costs of care for patients with relapsed and refractory Hodgkin lymphoma treated with brentuximab vedotin in the United States: A retrospective cohort study
Source: PLoS One. 2017 Oct 9;12(10):e0180261. doi: 10.1371/journal.pone.0180261 (PMC5633181; doi:10.1371/journal.pone.0180261)
Supplement: S1 Table — (DOCX) [file pone.0180261.s002.docx]

**S1 Table – HL-specific medications identified in the Truven MarketScan databases.**

| **Regimen name** | **Components (if applicable)** |
| --- | --- |
| ABVD | Adriamycin [doxorubicin], bleomycin, vinblastine, dacarbazine |
| BEACOPP | Bleomycin, etoposide, Adriamycin [doxorubicin], cyclophosphamide, Oncovin [vincristine], procarbazine, prednisone |
| Stanford V regimen | Doxorubicin, vinblastine, mechlorethamine, vincristine, bleomycin, etoposide, prednisone |
| C-MOPP | Cyclophosphamide, vincristine, procarbazine, prednisone |
| DHAP | Dexamethasone, high-dose cytarabine, cisplatin |
| ESHAP | Etoposide, steroid, cytarabine, cisplatin |
| GCD | Gemcitabine, carboplatin, dexamethasone |
| GVD | Gemcitabine, vinorelbine, pegylated liposomal doxorubicin |
| ICE | Ifosfamide, carboplatin, etoposide |
| IGEV | Ifosfamide with MESNA, gemcitabine, vinorelbine, prednisolone |
| BEAM | BCNU [carmustine], etoposide, cytarabine, melphalan |
| MINE | MESNA, ifosfamide, novantrone, etoposide |
| ChIVPP | Chlorambucil, vinblastine, procarbazine, prednisone |
| VIM-D | Etoposide, ifosfamide, mitoxantrone, dexamethasone |
| EPOCH | Doxorubicin, etoposide, cyclophosphamide, vincristine, prednisone |
| GDP | Cisplatin, dexamethasone, gemcitabine |
| ChLOPP | Vincristine, prednisone, procarbazine, chlorambucil |
| BELP | Etoposide, steroid, BCNU [carmustine], chlorambucil |
| CELP | Etoposide, steroid, chlorambucil, CCNU |
| MOPP | Vincristine, prednisone, procarbazine, mechlorethamine |
| Everolimus |  |
| Bortezomib |  |
| Alemtuzumab |  |
| Panobinostat |  |
| Chlorambucil |  |
| Brentuximab vedotin |  |
| Bendamustine |  |
| Gemcitabine |  |
| Rituximab |  |
| Lenalidomide |  |
