# Supplementary material for: Treatment patterns and costs of care for patients with relapsed and refractory Hodgkin lymphoma treated with brentuximab vedotin in the United States: A retrospective cohort study
Source: PLoS One. 2017 Oct 9;12(10):e0180261. doi: 10.1371/journal.pone.0180261 (PMC5633181; doi:10.1371/journal.pone.0180261)
Supplement: S2 Table — (DOCX) [file pone.0180261.s003.docx]

S2 Table ‒ Time from plan enrollment to BV initiation, and from BV initiation to censoring, in weeks.

|  | **Duration of therapy** | | | | | |
| --- | --- | --- | --- | --- | --- | --- |
|  | **Prior to BV initiation** | | **While receiving BV** | | **Post-BV discontinuation** | |
| **All BV (n=289)** |  |  |  |  |  |  |
| Mean (SD) | 180.6 | 118.9 | 22.7 | 23.8 | 48.5 | 36.5 |
| Median (IQR) | 153.9 | 73.3-281.4 | 15.3 | 6.1-30.9 | 39.7 | 22.3-68.7 |
| **BV in the line prior to ASCT (n=8)** |  |  |  |  |  |  |
| Mean (SD) | 201.8 | 155.9 | 7.9 | 8.4 | 68.2 | 37.2 |
| Median (IQR) | 179 | 65.4-326.6 | 6.6 | 2.4-9.4 | 56.6 | 48-75.2 |
| **BV salvage prior to ASCT (n=33)** |  |  |  |  |  |  |
| Mean (SD) | 181.3 | 132.9 | 17.2 | 20.2 | 58 | 44.7 |
| Median (IQR) | 158.3 | 55.7-289.3 | 9.1 | 4.1-18.1 | 41.6 | 27.4-76.1 |
| **BV post-ASCT relapse (n=91)** |  |  |  |  |  |  |
| Mean (SD) | 174.7 | 105.5 | 21.9 | 20 | 53.1 | 39.7 |
| Median (IQR) | 145.1 | 90.2-250.3 | 16.1 | 9.1-34.2 | 44.6 | 23.7-81.3 |
| **BV without observed ASCT (n=156)** |  |  |  |  |  |  |
| Mean (SD) | 183.4 | 122.2 | 25.1 | 26.7 | 42.8 | 31.7 |
| Median (IQR) | 155.4 | 70.8-305.5 | 18.3 | 6.6-33.1 | 37.1 | 16.9-58.9 |

BV, brentuximab vedotin; IQR, interquartile range; SD, standard deviation.
